# Expandable ELAST for super-resolution imaging of thick tissue slices using a hydrogel containing charged monomers

**DOI:** 10.1038/s41598-023-38891-3

**Published:** 2023-07-24

**Authors:** Woonggi La, Junyoung Seo, Eunseok Heo, Jae-Byum Chang

**Affiliations:** 1 Department of Materials Science and Engineering, Korea Advanced Institute of Science and Technology, Daejeon, 34141 South Korea; 2 Department of Biological Sciences, Korea Advanced Institute of Science and Technology, Daejeon, 34141 South Korea

**Keywords:** Cell biology, Neuroscience

## Abstract

Hydrogels have been utilized extensively as a material for retaining position information in tissue imaging procedures, such as tissue clearing and super-resolution imaging. Immunostaining thick biological tissues, however, poses a bottleneck that restricts sample size. The recently developed technique known as entangled link-augmented stretchable tissue-hydrogel (ELAST) accelerates the immunostaining process by embedding specimens in long-chain polymers and stretching them. A more advanced version of ELAST, magnifiable entangled link-augmented stretchable tissue-hydrogel (mELAST), achieves rapid immunostaining and tissue expansion by embedding specimens in long-chain neutral polymers and subsequently hydrolyzing them. Building on these techniques, we introduce a variant of mELAST called ExELAST. This approach uses charged monomers to stretch and expand tissue slices. Using ExELAST, we first tested two hydrogel compositions that could permit uniform expansion of biological specimens. Then, we apply the tailored hydrogel to the 500-μm-thick mouse brain slices and demonstrated that they can be stained within two days and imaged with a resolution below the diffraction limit of light.

## Introduction

Hydrogel engineering has attracted significant attention for its potential in solving problems in tissue imaging. It has been used for (1) tissue clearing^1–5^ and (2) super-resolution imaging via tissue expansion^6–10^. In tissue clearing, neutral monomer acrylamide has been used as scaffolding to preserve the positional information of proteins^1^. In tissue expansion techniques, charged monomer sodium acrylate has been used to expand tissues to improve image resolution. Expansion microscopy has achieved sub-diffraction limit resolution with diffraction-limited microscopy by homogeneously expanding biological specimens^6,7^.

In both tissue clearing and tissue expansion, the staining of thick biological specimens has been a bottleneck limiting the size of specimens that can be imaged. For example, 10-day immunostaining is needed to stain proteins inside whole coronal brain slices with a thickness of 1 mm^8^. To solve the problem of low antibody staining speed, a technique termed entangled link-augmented stretchable tissue-hydrogel (ELAST) has been proposed^11^. In this work, specimens are embedded in a hydrogel containing a low concentration of crosslinkers to enable the stretching of specimens. The stretching of specimens reduces their thickness, enabling much faster immunostaining of thick specimens. The same research group has recently proposed an improved version of ELAST termed magnifiable entangled link-augmented stretchable tissue-hydrogel (mELAST)^12^, which achieves both the stretching of specimens for rapid antibody staining and even the expansion of specimens for super-resolution imaging. In this work, neutral monomer acrylamide was used to achieve stretchability and hydrolyzed to achieve the expandability of specimens.

Here, we introduce expandable ELAST (ExELAST), which enables the stretching and expansion of specimens by forming hydrogels inside specimens using charged monomers. As poly-electrolyte hydrogels containing charged monomers are super-swellable, they are used in several expansion microscopy (ExM) techniques. To elucidate the ExELAST technique, we first optimize the hydrogel compositions and then show that specimens can be expanded and stretched using the optimized hydrogel composition. We then demonstrate the super-resolution imaging of thick brain slices by staining them while stretched and imaging them while expanded.

## Results

**Figure 1:**
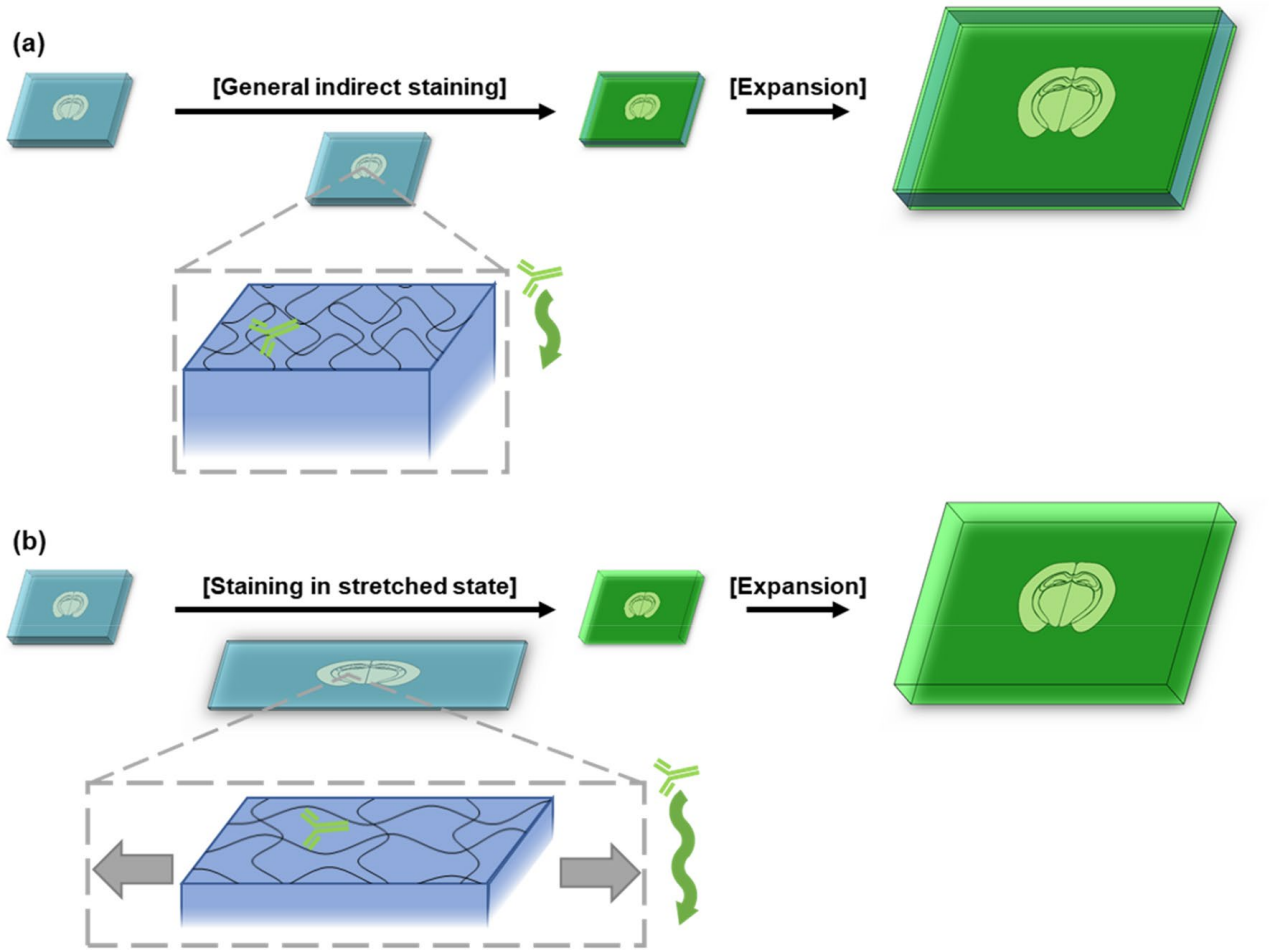
Schematic view of expandable ELAST. (**a**) Immunostaining without stretching only stains on the surface. (**b**) Stretched staining enables deeper immunostaining and expansion enables super-resolution imaging.

To determine candidates for hydrogel composition, we modified the hydrogel compositions used in protein-retention expansion microscopy (proExM)^7^. Since the first demonstration in 2016, proExM has been widely used in diverse applications, and its nanoscale expansion homogeneity has been validated in multiple studies. The proExM hydrogel is made up of 2.5% acrylamide (AAm), 7.5% sodium acrylate (SA), 0.15% crosslinker, 0.2% initiator, and 0.2% catalyst. To enable the stretching of specimens, we decreased the concentration of the crosslinker down to 0.02%, the initiator down to 0.05%, and the catalyst down to 0.05%. The use of low concentrations of the crosslinker, initiator, and catalyst enables the formation of long-chain polymers, which are needed for the stretching of specimens. We also increased the concentration of AAm to synthesize more tough hydrogels. The composition of the first hydrogel candidate was 15% AAm, 2.5% SA, 0.02% *N,Nʹ*-Methylenebisacrylamide (BIS), 0.05% initiator, and 0.05% catalyst. This hydrogel could be stretched more than twofold and expanded around fourfold in deionized water (DI). We also tested the second candidate, which contained an even lower concentration of crosslinker; for this candidate, a crosslinker concentration of 0.0075% was used. We adjusted the concentration of SA to achieve similar stretchability and expansion factors. The composition of the second hydrogel candidate was 11.5% AAm, 0.5% SA, 0.0075% BIS, 0.05% initiator, and 0.05% catalyst. Both of these candidate hydrogels enabled the stretching of biological specimens, such as brain slices, more than twofold. After embedding brain slices in these hydrogels, the specimens could be stretched, fixed at the stretched state, and then stained with antibodies to enable rapid antibody staining (Fig. 1).

**Figure 2:**
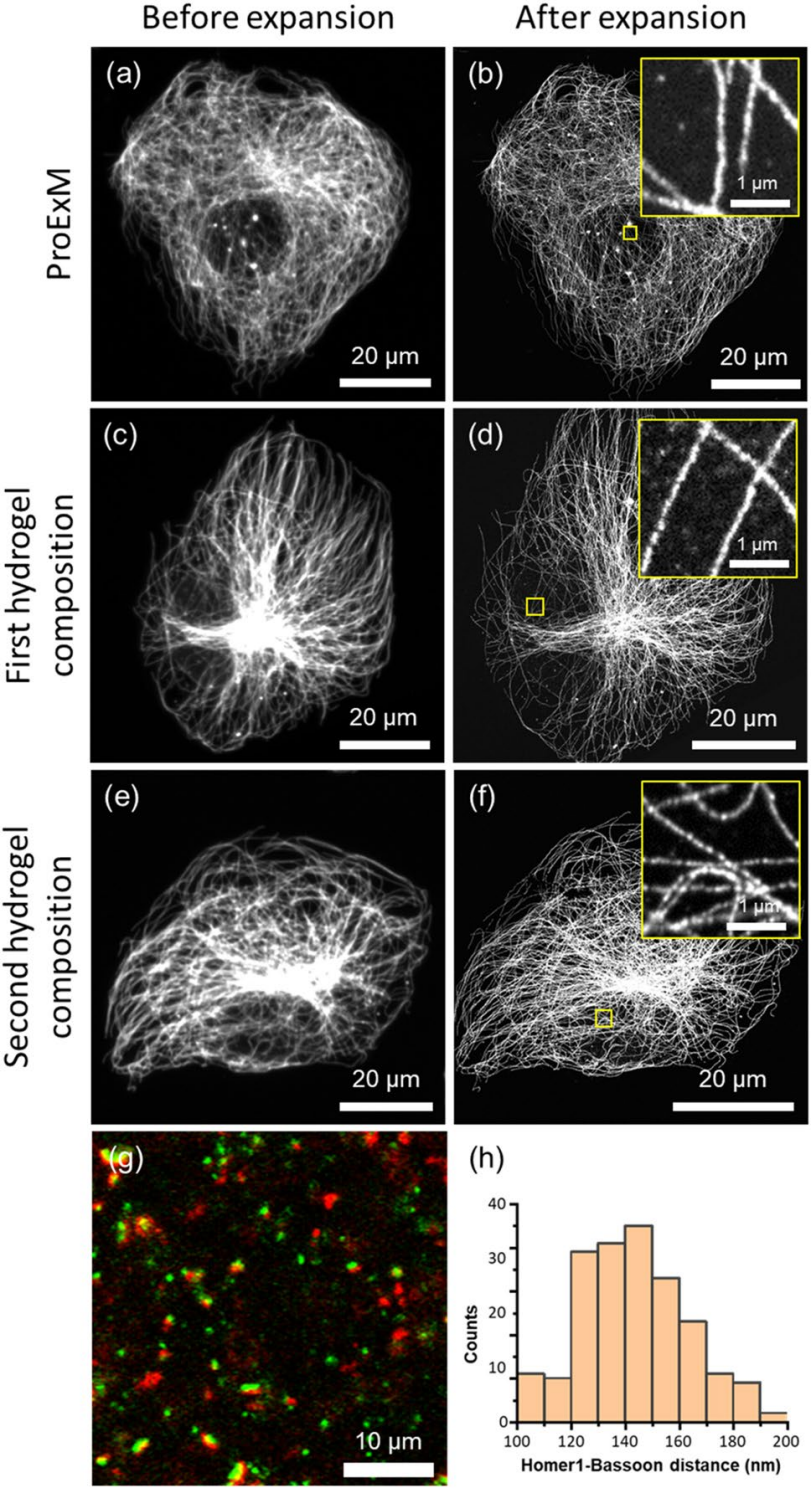
Microscopic distortion analysis of ExELAST strategy. (**a**–**f**) Tubulin of BS-C-1 cells was stained and imaged. (**a**,**b**) Before and after expansion image of proExM. (**c**,**d**) Before and after expansion image of the first hydrogel composition. (**e**,**f**) Before and after expansion image of the second hydrogel composition. (**g**) Pre-synaptic marker (Bassoon, red), and post-synaptic marker (Homer1, green) of mouse brain slices were stained and imaged with the first hydrogel composition. (**h**) Homer1-Bassoon distance profile of the expanded image. All the images were obtained with a 40× water immersion objective lens, 1.15 NA.

Of the two hydrogel compositions that enable both the stretching and expansion of specimens, we first tested which hydrogel better conserves nanoscale details. Cultured BS-C-1 cells were expanded using the following three hydrogel compositions: (1) the original proExM composition whose nanoscale expansion uniformity has been validated in multiple studies; (2) the first hydrogel candidate with a composition of 15% AAm, 2.5% SA, 0.02% BIS, 0.05% Ammonium persulfate (APS), and 0.05% *N,N,Nʹ,Nʹ*-Tetramethylethylenediamine (TEMED); and (3) the second hydrogel candidate with a composition of 11.5% AAm, 0.5% SA, 0.0075% BIS, 0.05% APS, and 0.05% TEMED. Microtubules of BS-C-1 cells were stained with an anti-tubulin antibody and fluorophore-conjugated secondary antibody. Then, the cells were treated with 6-((acryloyl)amino)hexanoic Acid, succinimidyl ester (AcX), which makes all proteins, including the antibodies, gel-anchorable. After that, the cells were incubated in one of three hydrogel solutions, followed by gelation and proteinase K digestion. Then, the hydrogels were expanded in DI water, and the microtubules were imaged using confocal microscope. Figure 2a,c,e show representative images of the microtubules after immunostaining. Figure 2b,d,f show confocal microscope images of the same cells shown in Figs. 2a,c,e, expanded by one of the three hydrogel compositions. The expansion ratio of the proExM was 3.97, showing good agreement with the literature^7^ (Fig. 2a,b). The expansion ratio of the first and second candidates was 4.67 (Fig. 2c,d) and 4.76, (Fig. 2e,f), respectively. While proExM hydrogel and the first candidate showed improvement in the resolution of the image without notable distortion, the second candidate showed signal intensity variation along microtubules, suggesting that the hydrogel's expansion was not uniform (Fig. 2f, Supplementary Fig. 1). Based on these findings, we chose the first hydrogel composition as a ExELAST hydrogel.﻿

We then used the ExELAST hydrogel composition to form an expandable hydrogel inside mouse brain slices, expanded them, and measured the microscopic expansion uniformity. The identical locations from the pre- and post-expansion images were randomly selected. The distances between identical locations were measured from pre- and post-expansion images and recorded as pairs. A total of 30 pairs of distances from three different samples were measured and linearly regressed. For the 0.02× phosphate-buffered saline (PBS) expanded samples, the fitted slope was 2.94, and the R squared was 0.99828, with a fixed intercept of 0. The 0.02× PBS expanded samples also showed a narrow distribution between each expansion factor without notable sample-to-sample variation (Supplementary Fig. 2). The DI expanded samples shown in Supplementary Fig. 3 showed a broader distribution than the 0.02× PBS expanded samples. While the fitted slope was 3.79 with an R squared of 0.99798, a broader distribution with considerable sample-to-sample variation was observed. Based on these results, we expanded the specimens in 0.02× PBS with an expansion factor of 2.94 in subsequent experiments to better preserve the microscopic structures.

Next, we validated the nanoscopic expansion uniformity using mouse brain slices. The slices were embedded in the ExELAST hydrogel, denatured, and stained with antibodies against pre-synaptic marker (Bassoon) and the post-synaptic marker (Homer1). The slices were then expanded, and the distance between Homer1 and Bassoon was measured and compared with the previous literature^6,13,14^. Figure 2g shows representative images of the pre- and post-synaptic markers after 0.02× PBS expansion. The image showed good antibody specificity without any non-specific bindings. The image also shows the signal separation of the pre-synaptic marker and the post-synaptic marker , which supports the high resolution of ExELAST. The distance between Homer1 and Bassoon was measured in 225 pairs from three different samples. The average distance between Homer1 and Bassoon was 143.8 ± 19.7 nm, which shows good agreement with previously reported data produced using other super-resolution imaging techniques (Fig. 2h). Based on this result, we concluded that ExELAST hydrogel effectively conserves nanoscale details.

We then applied the ExELAST strategy to various antibodies to test whether it could be used for the super-resolution imaging of proteins inside thick brain slices. Mouse brain slices with a thickness of 150 μm were incubated in an ExELAST hydrogel solution, proceeded by gelation. The hydrogel–brain slice composites were treated in a denaturation buffer at 85 °C to enable the uniform expansion of the slices without any harsh proteinase treatment by breaking the inter-protein interactions^8,9^. After denaturation, the hydrogel-brain composites were stained with antibodies against 30 targets, including the following: synaptic proteins (vesicular glutamate transporter 1 (vGluT1), homer1, bassoon, and piccolo); cell-type markers (calcium binding protein 1 (CALB1), and glial fibrillary acidic protein (GFAP)); myelin structure markers (myelin basic protein (MBP), 2ʹ,3ʹ-cyclic nucleotide 3ʹ-phosphodiesterase (CNP1)); and cytoskeleton-related proteins (microtubule-associated protein 2 (MAP2), tubulin, and vimentin). Among the 30 targets tested, 20 were successfully stained after the embedding of the mouse brain slices in the ExELAST hydrogel (Supplementary Fig. 4). While ExELAST conserves nanoscale details with various available antibodies, some protein structures exhibited uneven signals after expansion. Specifically, collagen-rich structures, such as blood vessels, showed variations in fluorescent signal intensities along their structures (Supplementary Fig. 5a,d). To address this issue, we initially attempted a milder fixation approach and used 3% paraformaldehyde (PFA) instead of 4% PFA (Supplementary Fig. 5b,e). However, this adjustment did not resolve the intensity variation issues in the blood vessels. We then introduced an enzymatic digestion step. After 37 °C sodium dodecyl sulfate (SDS)-based clearing, the specimens were treated with a collagenase cocktail and stained with an antibody against glucose transporter 1 (GluT1). The inclusion of collagenase treatment significantly alleviated the intensity variation problem, resulting in more uniform fluorescence signal intensities along the blood vessels (Supplementary Fig. 5c,f).

**Figure 3:**
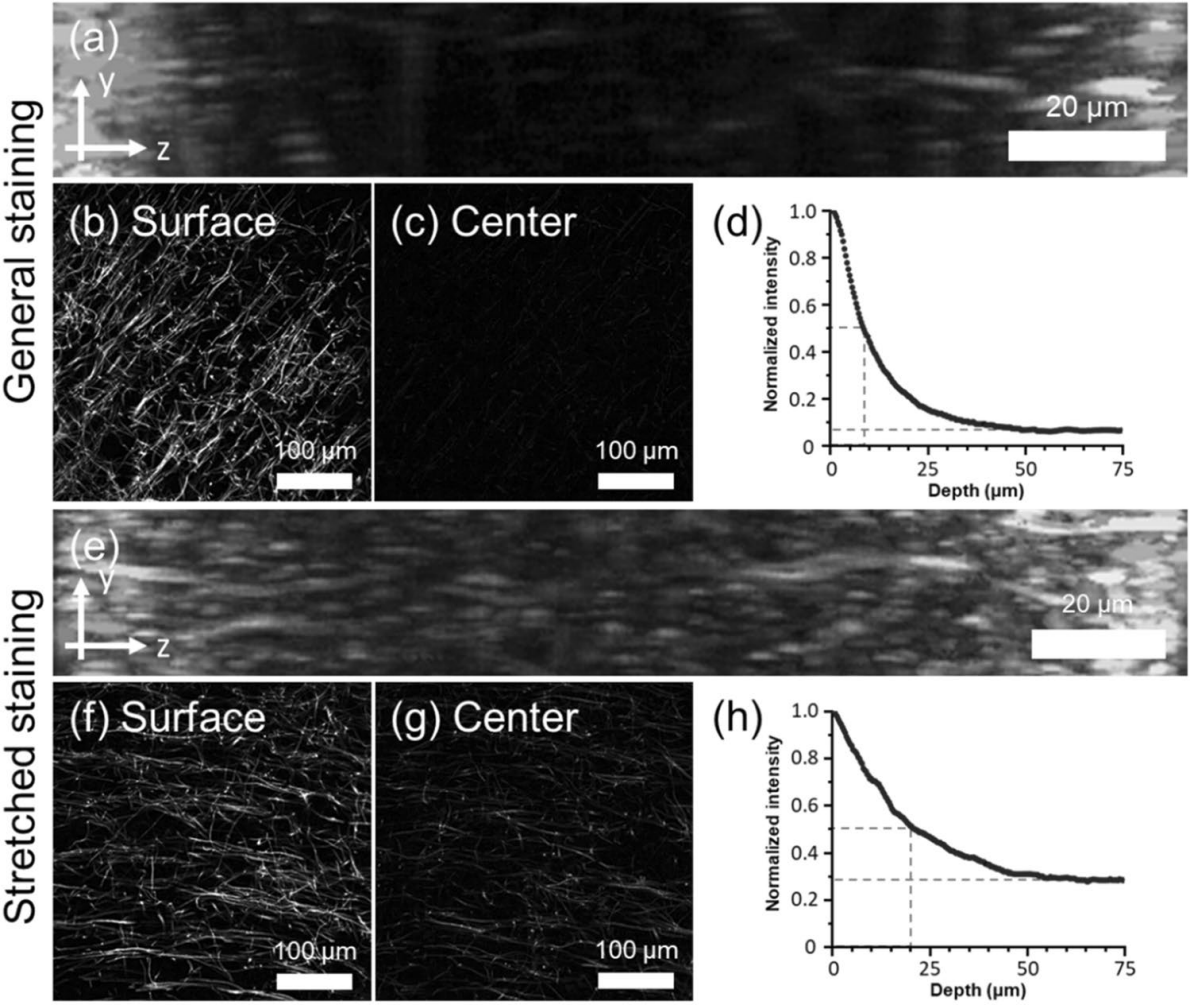
A comparison of immunostaining depth in non-stretched state and stretched state of ExELAST hydrogel. Myelin Basic Protein (MBP) of mouse brain slices was stained and imaged. For (**a–d**), staining was performed without stretching. (**a**) Side view of MBP single channel z-stack image using 3D projection function of Fiji software. (**b**) Myelin sheath image acquired at the surface. (**c**) Myelin sheath image acquired at the center of the depth. (**d**) Normalized intensity of top 0.5% brightest pixels of each slice for MBP channel. For (**e–h**), staining was performed without stretching. (**e**) Side view of MBP single channel z-stack image using 3D projection function of Fiji software. (**f**) Myelin sheath image acquired at the surface. (**g**) Myelin sheath image acquired at the center of the depth. (**h**) Normalized intensity of top 0.5% brightest pixels of each slice for MBP channel. All the images were obtained with a 10× dry objective lens, 0.45 NA.

Once the expansion factor and antibody availability of the ExELAST hydrogel was confirmed, we then performed the rapid immunostaining of the brain slices using this hydrogel. Two brain slices with a thickness of 150 μm were embedded in the ExELAST hydrogel and denatured. After denaturation, the first slice was incubated in a solution containing myelin basic protein-targeting antibody for 4 h. The second slice was stretched twofold and then incubated in the same solution. Following the primary antibody staining, the first brain slice was stained with secondary antibodies without any stretching for 4 h. The second brain slice was stained with secondary antibodies, again in a stretched state. We then acquired z-stack images of these two slices from the bottom to the top surfaces. As shown in Fig. 3a–d and Supplementary Fig. 6a, the first brain slice, which was stained with antibodies without any stretching, shows non-uniform staining intensities. MBP showed strong fluorescence intensity on the surfaces but only about 6% of that intensity at the mid-plane of the brain slice. The second brain slice, which was stained with antibodies while being stretched twofold, showed more uniform fluorescence intensity along the z-axis (Fig. 3e–h, Supplementary Fig. 6b). MBP showed about 28% of the fluorescence intensity on the surfaces at the mid-plane of the brain slice. Whereas the unstretched sample reached half-maximum intensity at a depth of 8 µm, the stretched sample reached half-maximum intensity at a depth of 20 µm. We also tested immunostaining with a larger staining volume and a high salt concentration. Although the hydrogel was thinner due to the high salt concentration, there was no notable increase in immunostaining speed from the normalized intensity profile (Supplementary Fig. 6c). Similarly, a large volume of staining buffer used to maintain the concentration gradient did not notably increase immunostaining speed (Supplementary Fig. 6d). This result shows that the immunostaining of brain slices could be accelerated by embedding the brain slices in an ExELAST hydrogel and stretching them.

**Figure 4:**
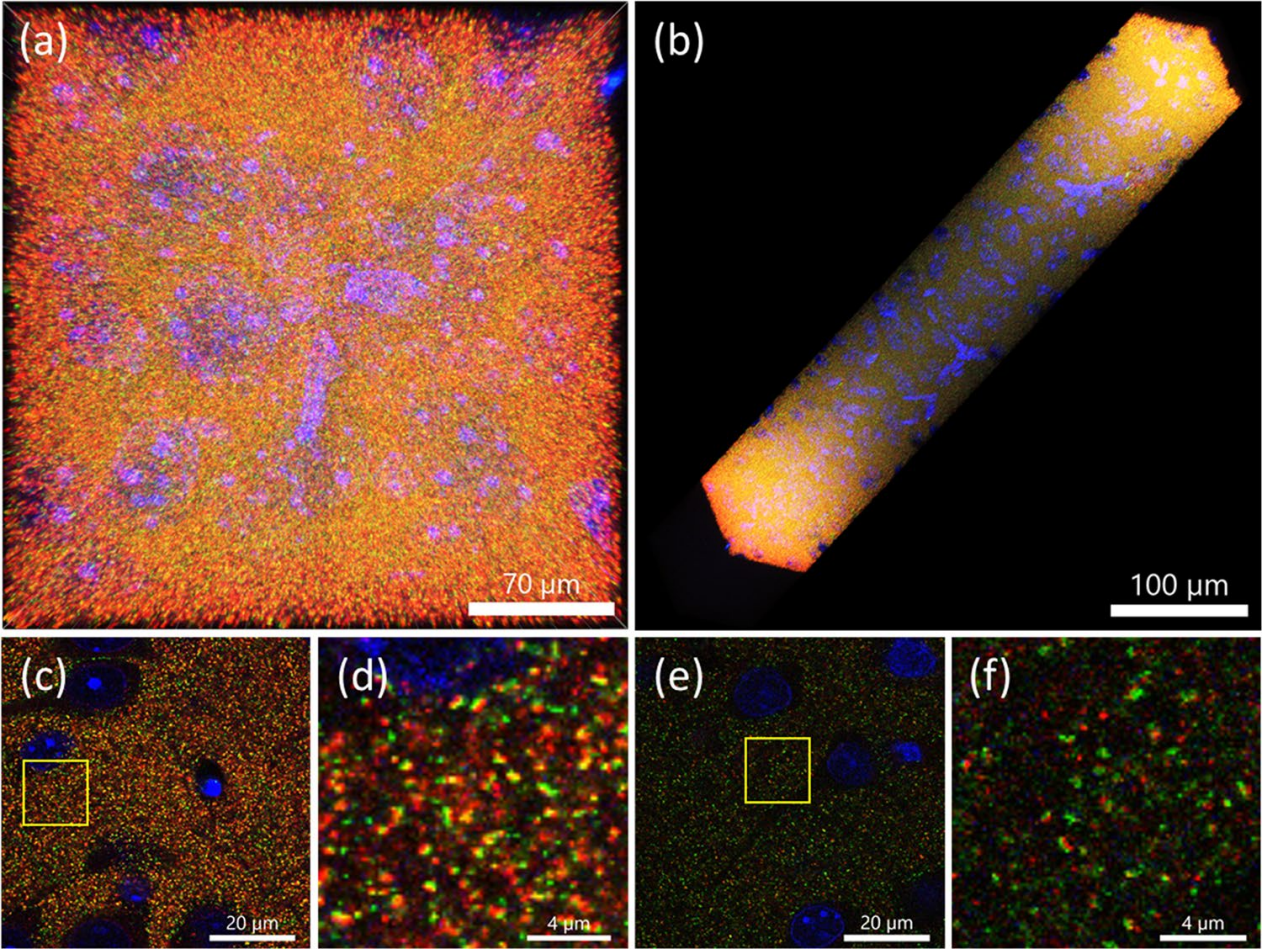
Immunostaining of thick mouse brain slices through ExELAST. (**a**,**b**) Maximum intensity projection (MIP) image of fully stained 500-μm-thick mouse brain slice. (**c**) Image acquired at the surface. (**d**) Enlarged image of yellow boxed region in **c**. (**e**) Image acquired at the center of the depth. (**f**) Enlarged image of yellow boxed region in **e**. Blue: 4′,6-diamidino-2-phenylindole dihydrochloride (DAPI), red: Homer1, green: Bassoon. All the images were obtained with a 60× water immersion objective lens, 1.00 NA.

Finally, we applied the ExELAST hydrogel to a brain slice and demonstrated the staining of the whole brain slice and super-resolution imaging. A brain slice with a thickness of 500 μm was embedded in ExELAST hydrogel, denatured, stretched twofold, and stained with primary antibodies against two synaptic marker proteins (Bassoon and Homer1) and secondary antibodies. Although the slice was stained with antibodies for only 2 days, the synapses inside the 500-μm-thick brain slice were stained. As shown in Fig. 4, the super-resolution level structures of the synapse, such as the two-separated signals representing pre-synaptic protein and post-synaptic protein, were clearly shown from the surface to the mid-plane of the brain slice.

## Discussion

In this work, we demonstrated that ExELAST enables both the stretching and expansion of biological specimens. We chose two hydrogel compositions containing charged monomers and a crosslinker at a concentration lower than that of the hydrogel used in proExM. The use of a low crosslinker concentration enabled the stretching of the specimens, and the use of charged monomers enabled the expansion of the specimens. We first tested ExELAST hydrogel could conserve nanoscale details. The pre- and post- synaptic markers of mouse brain slices expanded via ExELAST hydrogel showed similar distance profiles with others, indicating that the expansion uniformity of ExELAST is similar to previous super-resolution techniques^6,13,14^. Then, we showed that ExELAST-processed brain slices can be stained with a variety of antibodies. By embedding brain slices in the ExELAST hydrogel and staining them while stretched, a more uniform staining profile along the thickness of the slices was achieved. Finally, we demonstrated the super-resolution imaging of synaptic markers in a 500-μm-thick brain slice by staining it with antibodies while stretched and acquiring images after expansion.

The ExELAST demonstrated in this work could be improved in multiple aspects. Recently, diverse hydrogel compositions enabling the expansion of specimens more than fourfold have been reported^14–16^. Such ExM techniques with larger expansion factors enable the super-resolution imaging of specimens with a resolution higher than 60 nm. New hydrogel compositions that enable the expansion of specimens more than fourfold and the stretching of specimens warrant further exploration and examination. In keeping with this, the relevant denaturation conditions would also require further study. In the present work, 20 out of 30 antibodies stained the brain slices after the specimens were embedded in the ExELAST hydrogel. Further optimization of the denaturation conditions is required to enable the application of a wider range of antibodies and the expansion of diverse non-soft tissues, such as the heart and liver. Finally, the combination of ExELAST with multiplexed imaging techniques, such as the DNA-based multiplexing^17^ or spectral unmixing^18^, would enable the simultaneous imaging of more than 10 proteins over thick specimens.

Furthermore, ExELAST would be a useful tool for the super-resolution imaging of a large volume. Recently, the use of ExM has been expanded to whole mouse embryos^19^. In imaging such large specimens, antibody staining is the biggest issue. When combined with whole-body ExM, ExELAST would enable the three-dimensional super-resolution imaging of specific proteins over entire vertebrates.

## Methods

All materials used in this study are listed in Supplementary Tables 1 and 2.

### Cell culture and preparation

BS-C-1 cell was purchased from the Korea Cell Line Bank. Cell culture medium was prepared by diluting 100 U/mL penicillin–streptomycin, 1 mM sodium pyruvate, and 10% fetal bovine serum in a minimum essential medium. BS-C-1 cells were cultured on microscope cover glass (Marienfeld, 0111520) in 24 well cell culture plates (SPL, 30024) using a prepared cell culture medium at 37 ℃ in humified 5% CO_2_. Cultured cells were washed three times with 1× PBS briefly and fixed for 10 min with 3% paraformaldehyde and 0.1% glutaraldehyde in 1× PBS. The cells were incubated with 0.1% sodium borohydride in 1× PBS for 7 min, followed by washing with 1× PBS for 5 min three times. All of the processes were performed using the ice-cold solution at room temperature (RT).

### Cell staining

Fixed cells were blocked and permeabilized in NGS blocking buffer (0.1% Triton X-100 and 5% normal goat serum in 1× PBS) for 1 h. The blocked cells were incubated with the primary antibody in NGS blocking buffer for 1 h, followed by washing with NGS blocking buffer for 10 min three times. The cells were then incubated with secondary antibody in NGS blocking buffer for 1 h, followed by washing with NGS blocking buffer for 10 min three times. All of the processes were performed under RT.

### Cell gelation and expansion

The stained cells were washed with PBST (0.1% Triton X-100 in 1× PBS) for 10 min three times. The washed cells were incubated with 0.1 mg/mL 6-((acryloyl)amino)hexanoic Acid, succinimidyl ester (AcX) in PBST overnight, followed by washing with PBST for 10 min three times. The anchored cells were incubated in gel solution (proExM: 2.5% AAm, 7.5% SA, 0.15% BIS, 0.2% APS, 0.2% TEMED, 0.01% 4-hydroxy-TEMPO (H-TEMPO), 2 M sodium chloride (NaCl) in 1× PBS, First candidate: 15% AAm, 2.5% SA, 0.02% BIS, 0.05% APS, 0.05% TEMED, in 1× PBS, Second candidate: 11.5% AAm, 0.5% SA, 0.0075% BIS, 0.05% APS, 0.05% TEMED, in 1× PBS. All percentages; w/w) for 10 min and initiated gelation for 3 h at 37 ℃ under humid nitrogen conditions. All of the processes before thermo-initiation were performed at 4 ℃. After gelation, cell-hydrogel complexes were incubated in digestion buffer (8 units/mL Proteinase K, 1 M NaCl, 1 mM ethylenediaminetetraacetic acid (EDTA), 0.5% Triton X-100 and 50 mM tris(hydroxymethyl)aminomethane hydrochloride (Tris–HCl) in deionized water (DI)) overnight, followed by washing with PBST for 30 min three times. Digested hydrogels were then stained with DAPI in PBST for 1 h, and expanded in an excessive amount of DIW with several times of solvent changes. All of the processes were performed under RT.

### Antibody: fluorophore conjugation and purification

10 μL of 1 M sodium bicarbonate (pH 8.3) was added to 90 μL of antibody solutions to make Alexa Fluor 546 conjugated donkey anti-guinea pig antibody and Alexa Fluor 488 conjugated donkey anti-rabbit antibody. Then, a ninefold molar excess of Alexa Fluor 546 or Alexa Fluor 488 NHS ester stock (10 mg/mL) was added to the solution, and the solutions were reacted at RT for 1 h in the dark state. 10 mL of 1× PBS was loaded into NAP-5 gel filtration columns for equilibration and then the reacted antibody-fluorophore solutions were loaded into the columns. After adding 900 μL of 1× PBS to the columns, the eluates containing fluorophore-conjugated antibodies were collected. Following that, centrifugal filters with a molecular weight cut-off (MWCO) of 50,000 were used to concentrate the eluates.

### Brain preparation

All of the processes related to animals were approved by the Korea Advanced Institute of Science and Technology Institutional Animal Care and Use Committee (KAIST-IACUC) and conducted the study in compliance with the guidelines and regulations of the same organization. Mice were maintained in a specific pathogen-free facility of the KAIST Laboratory Animal Resource Center. C57BL/6J mice aged 6–12 weeks were used. Mice were inhalational anesthetized with isoflurane and transcardially washed with ice-cold 1× PBS followed by 4% paraformaldehyde in 1× PBS. Mouse brains were harvested and fixed with 4% paraformaldehyde in 1× PBS at 4 ℃ for 2–4 h followed by washing in 1× PBS at 4 ℃ overnight. Fixed brains were sliced into 150-μm or 500-μm-thick with a vibratome (Leica, VT1000S) and stored in a storage solution (0.1 M glycine and 0.01% sodium azide in 1× PBS) at 4 ℃.

### Brain gelation and denaturation

All of the processes in brain gelation and expansion were performed with gentle shaking. 150-μm-thick fixed brains were anchored with AcX in PBST overnight and washed with PBST for 30 min three times. Anchored brain slices were incubated in gel solution for 30 min at 4 ℃ two times, then initiated gelation for 3 h at 37 ℃ under humid nitrogen conditions. For 500-μm-thick brain slices, washing and monomer diffusion steps were performed twice longer. After gelation, 150-μm-thick tissue-hydrogel complexes were incubated in the clearing buffer (6% SDS, 50 mM sodium sulfate, and 0.02% sodium azide in 1× PBS) at 37 ℃ overnight. Cleared tissue-hydrogel complexes were then denatured in the pre-heated clearing solution for 6 h at 85 ℃, followed by washing with PBST for 30 min at 37 ℃ three times. For 500-μm-thick brain samples, washing steps were performed twice longer. For collagenase-treated samples, 37 ℃ SDS-cleared tissue-hydrogel complexes were digested with 0.2 mg/mL collagenase 1, 2, and 4 cocktail in 1× Hank's balanced salt solution (HBSS) overnight, followed by washing with PBST for 30 min at RT.

### Brain staining

Tissue-gel composites were stretched manually. Left and right ends of the hydrogel were attached to two holding glasses with superglue and then stretched around twofold. The stretched hydrogel was attached to the slide glass, maintaining the stretched state. Denatured tissue–hydrogel complexes were blocked and permeabilized in NGS blocking buffer overnight. Blocked 150-μm thick tissue-hydrogel complexes were then incubated with the primary antibody in NGS blocking buffer for 4 h, followed by washing with NGS blocking buffer for 30 min three times. The complexes were then incubated with the secondary antibody in NGS blocking buffer for 4 h. As for the 500-μm-thick brain samples, antibody staining was performed for two days, and the washing steps were performed twice as long. Stained tissue-hydrogel complexes were expanded in an excessive amount of 0.02× PBS solution with several times of solvent changes. All of the processes were performed under RT.

### Sample mounting and imaging

Expanded hydrogels were attached to 0.1% poly-l-lysine treated coverglass to prevent drifting during imaging. Samples were imaged with a spinning disk confocal microscope (Andor, Dragonfly 200).

## Supplementary Information

Supplementary Information.

## Data Availability

The datasets used and/or analyzed during the current study are available from the corresponding author upon reasonable request.
