# Supplementary material for: Expandable ELAST for super-resolution imaging of thick tissue slices using a hydrogel containing charged monomers
Source: Sci Rep. 2023 Jul 24;13:11929. doi: 10.1038/s41598-023-38891-3 (PMC10366192; doi:10.1038/s41598-023-38891-3)
Supplement: Supplementary file 1 — Supplementary Information. [file 41598_2023_38891_MOESM1_ESM.docx]

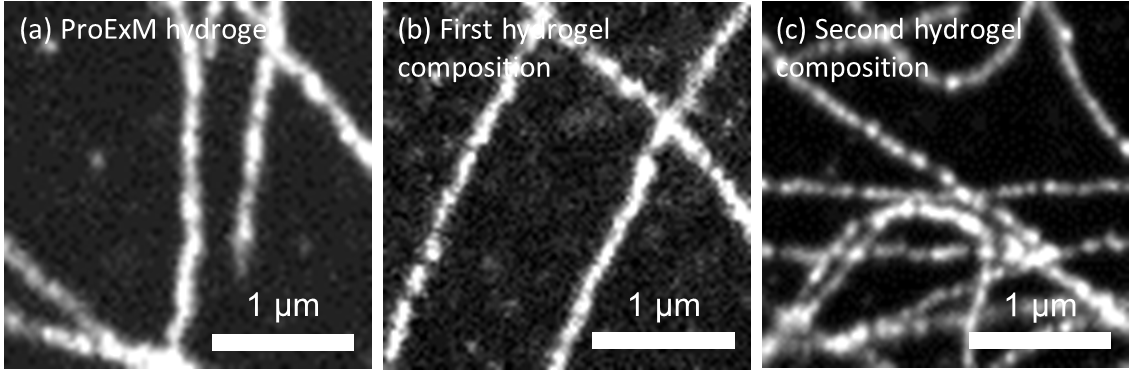


**Supplementary Figure 1**. Enlarged microtubule images expanded with three different hydrogels. Microtubules of BS-C-1 cells are stained and imaged. (**a**) Microtubule image expanded with ProExM hydrogel. (**b**) Microtubule image expanded with first hydrogel composition. (**c**) Microtubule image expanded with second hydrogel composition. All the images were obtained with 40× water immersion objective lens, 1.15 NA.


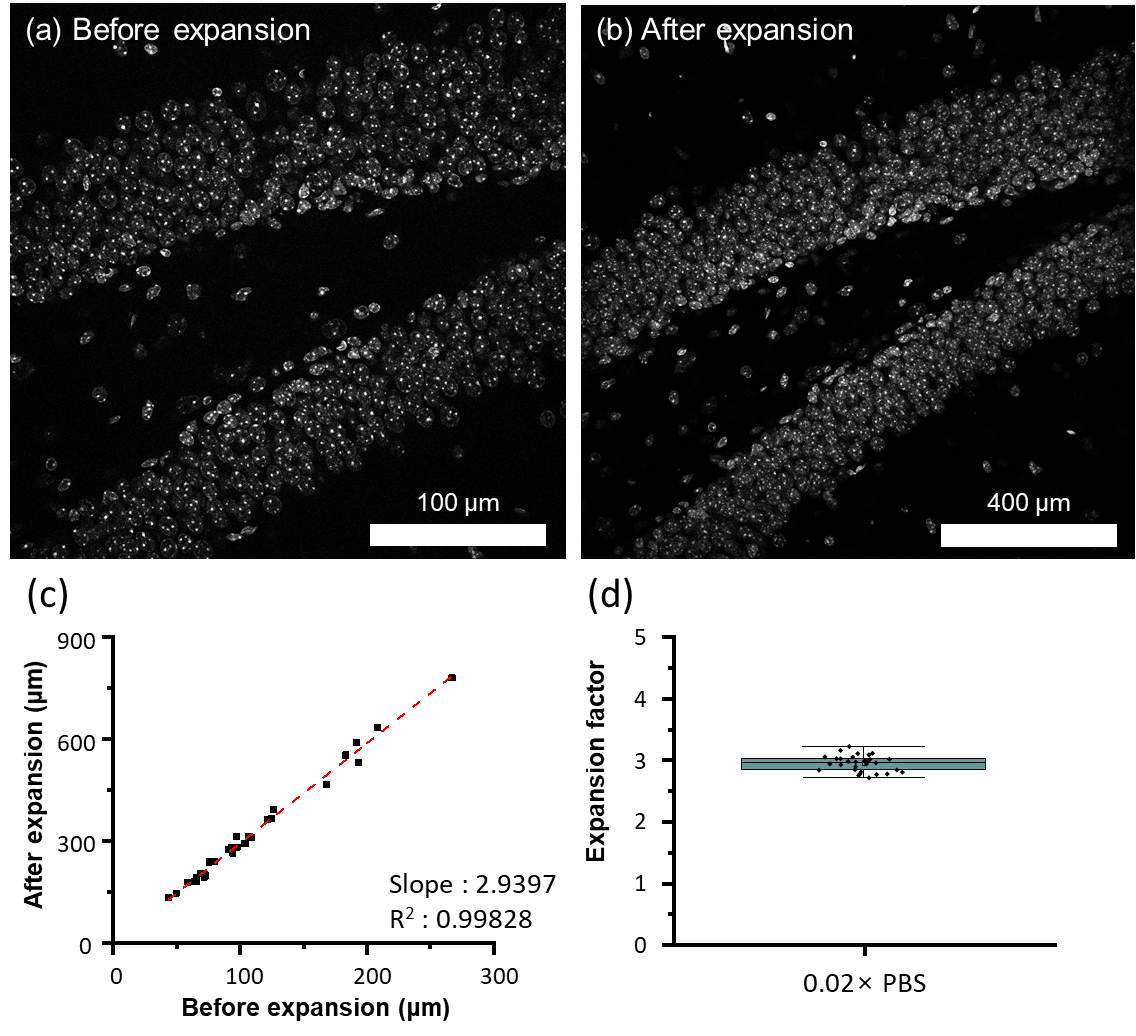


**Supplementary Figure 2**. Expansion factor analysis for 0.02× PBS expanded samples. (a) Before expansion image of DAPI single channel. (b) After expansion image of DAPI single channel. The scale bar is not corrected through expansion factor. (c) Linear regression analysis for 0.02× PBS expanded samples through pre-and-post expansion DAPI signal distances. (d) Distribution analysis of expansion factors for 0.02× PBS expanded samples. Standard deviation was 0.131 and median was 2.96. 1^st^ Quartile (upper boundary of colored box) was 2.83 and 3^rd^ Quartile (lower boundary of colored box) was 3.02. Maximum was 3.22 and minimum was 2.71. Before expansion image was obtained with 40× water immersion objective lens, 1.15 NA. After expansion image was obtained with 10× dry objective lens, 0.45 NA


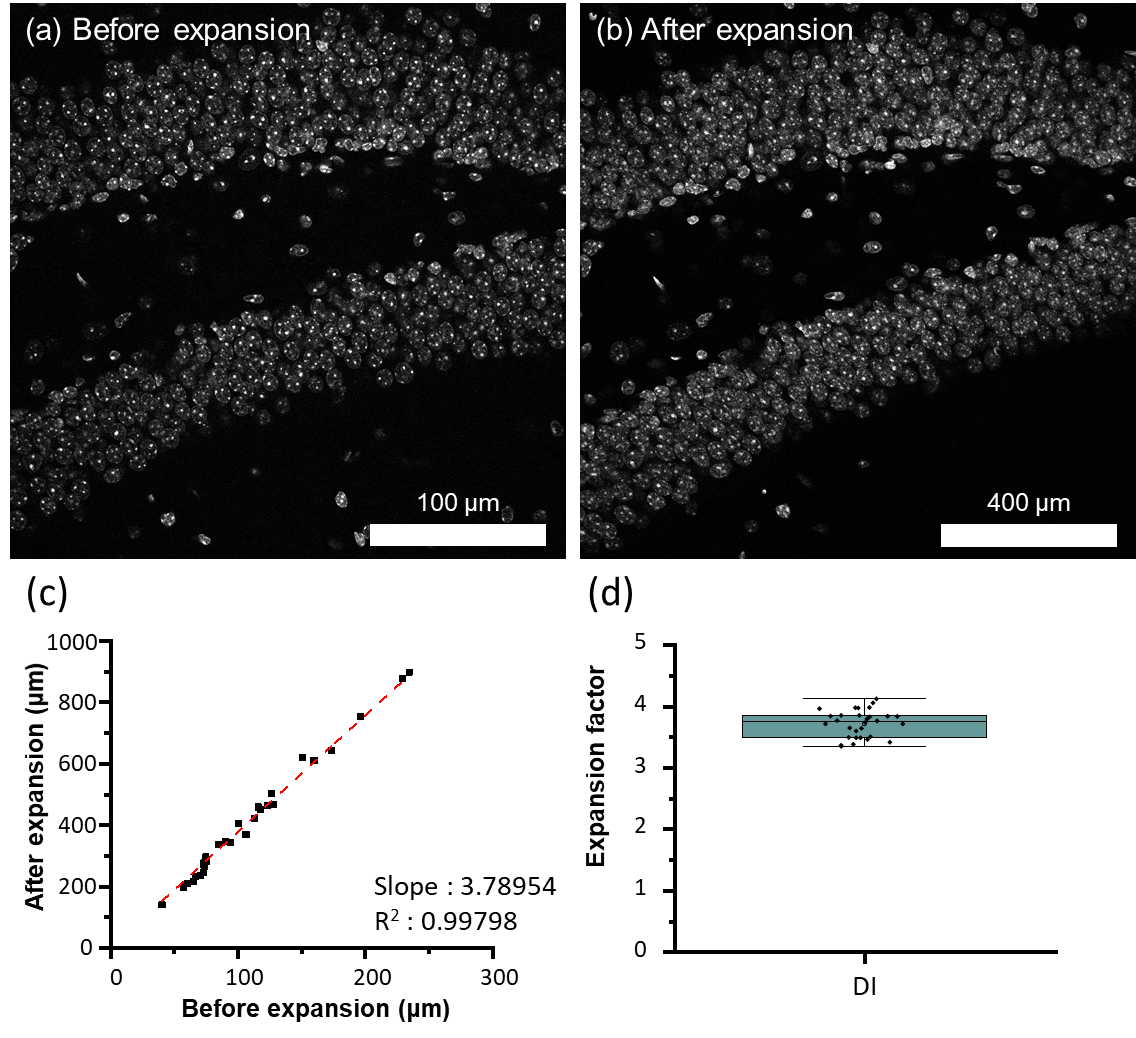


**Supplementary Figure 3**. Expansion factor analysis for DI expanded samples. (a) Before expansion image of DAPI single channel. (b) After expansion image of DAPI single channel. The scale bar is not corrected through expansion factor. (c) Linear regression analysis for DI expanded samples through pre-and-post expansion DAPI signal distances. (d) Distribution analysis of expansion factors for DI expanded samples. Standard deviation was 0.220 and median was 3.75. 1^st^ Quartile (upper boundary of colored box) was 3.49 and 3^rd^ Quartile (lower boundary of colored box) was 3.85. Maximum was 4.12 and minimum was 3.34. Before expansion image was obtained with 40× water immersion objective lens, 1.15 NA. After expansion image was obtained with 10× dry objective lens, 0.45 NA

**
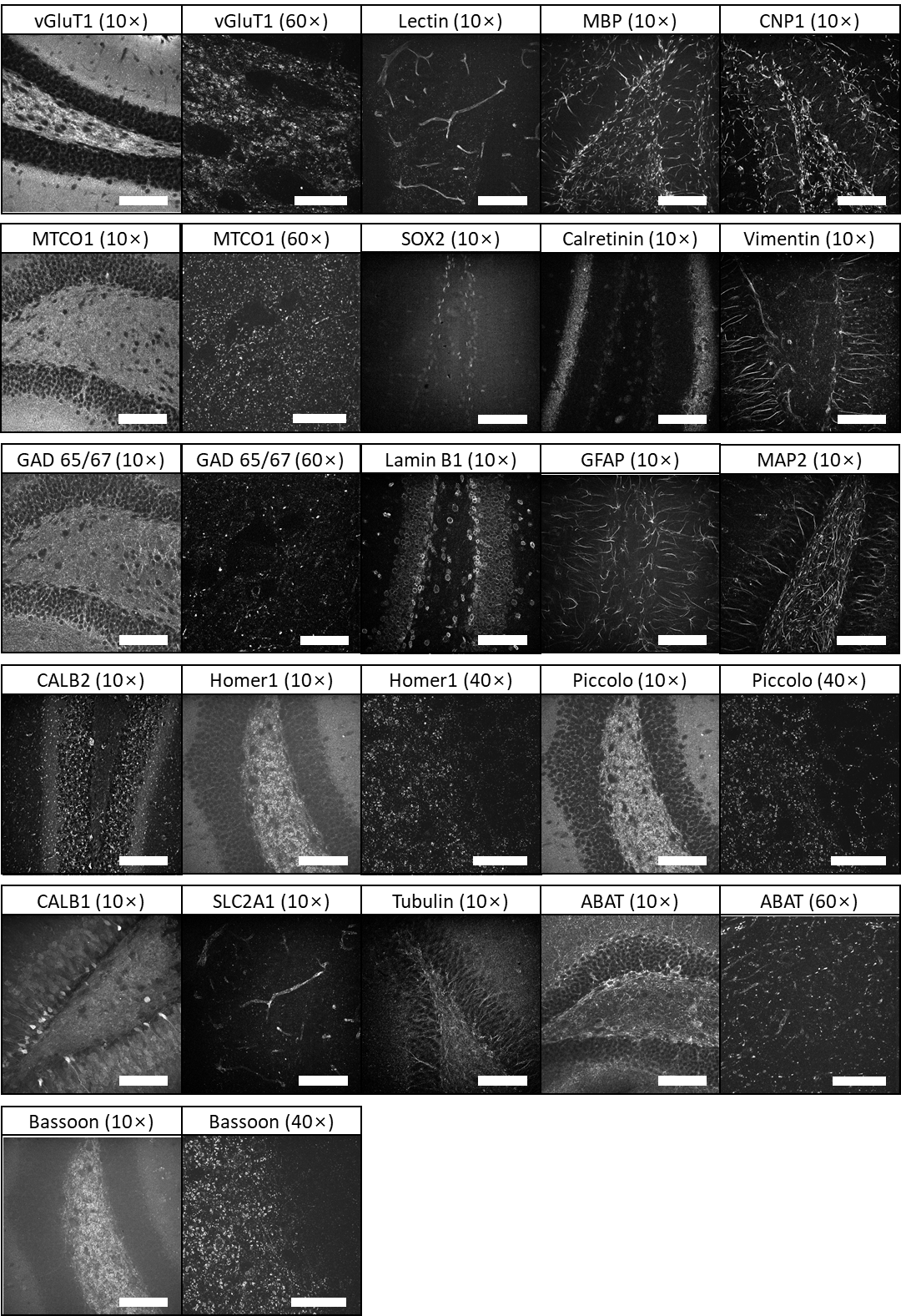
**

**Supplementary Figure 4**. Antibody screening test for ExELAST. All the 10× images were obtained with 10× dry objective lens, 0.45 NA. All scale bars for 10× images; 100µm. Synaptic markers and mitochondria markers are also obtained with higher magnification lens. 40× images were obtained with 40× water immersion objective lens, 1.15 NA. All scale bars for 10× images; 25µm. 60× images were obtained with 60× water immersion objective lens, 1.00 NA. All scale bars for 60× images; 15µm.

**
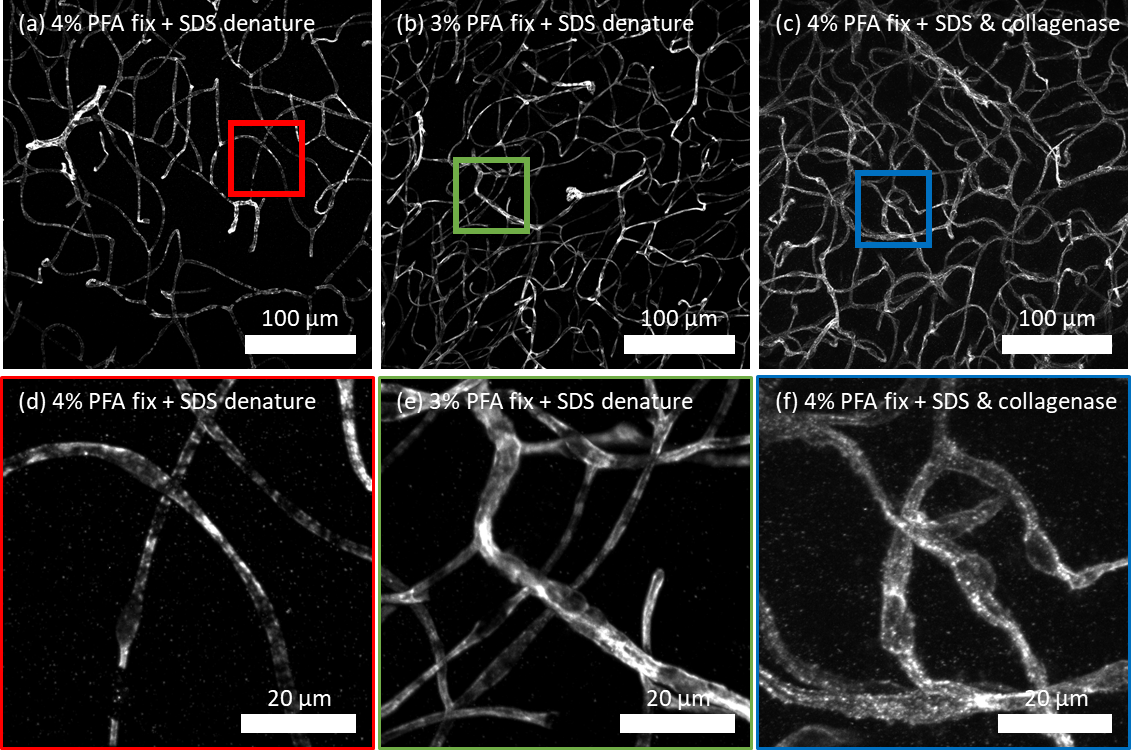
**

**Supplementary Figure 5**. Blood vessel images after expansion. Maximum intensity projection image of mouse brain slices. Glucose transporter type 1 is stained and imaged. (**a**) Blood vessel image of ExELAST-processed sample. (**b**) Blood vessel image of 3%-PFA-fixed ExELAST-processed sample. (**c**) Blood vessel image of additional collagenase-treated samples. (**d**) Enlarged image of red box in (**a**). (**e**) Enlarged image of green box in (**b**). (**f**) Enlarged image of red box from (**c**). All images were obtained with 10× dry objective lens, 0.40 NA

**
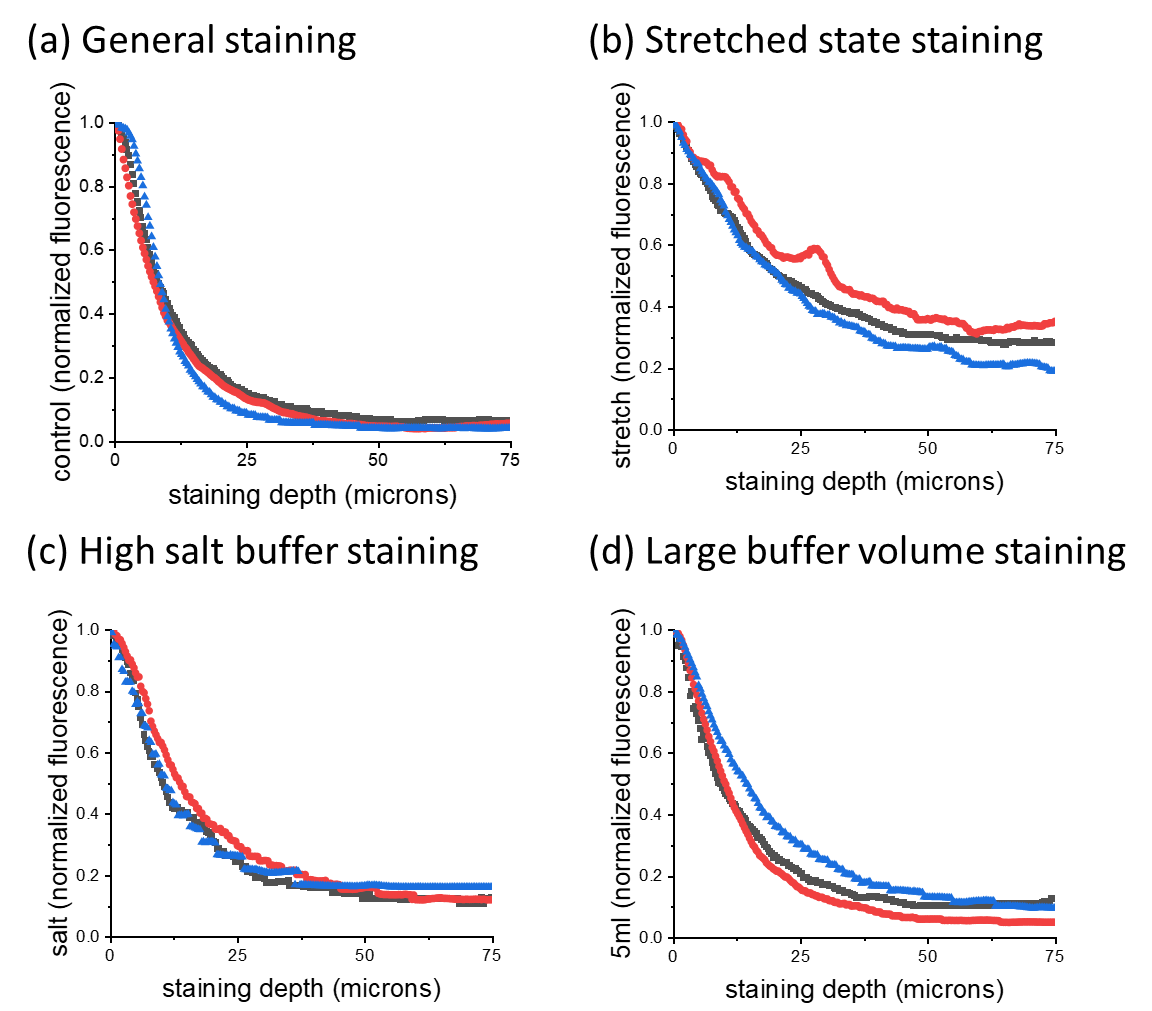
**

**Supplementary Figure 6**. Intensity profile from immunostaining depth data depending on stretching. Myelin Basic Protein is stained in several conditions and imaged for each of the 3 samples. Normalized intensity is calculated for the top 0.5% brightest pixels of each slice for the MBP channel. **(a**) Denatured gel-tissue hybrid is stained in a general way. (**b**) Denatured gel-tissue hybrid is stained while maintaining stretched. (**c**) Denatured gel-tissue hybrid is stained with staining buffer containing high salt concentration. (**d**) The denatured gel-tissue hybrid is stained with a larger volume of staining buffer.


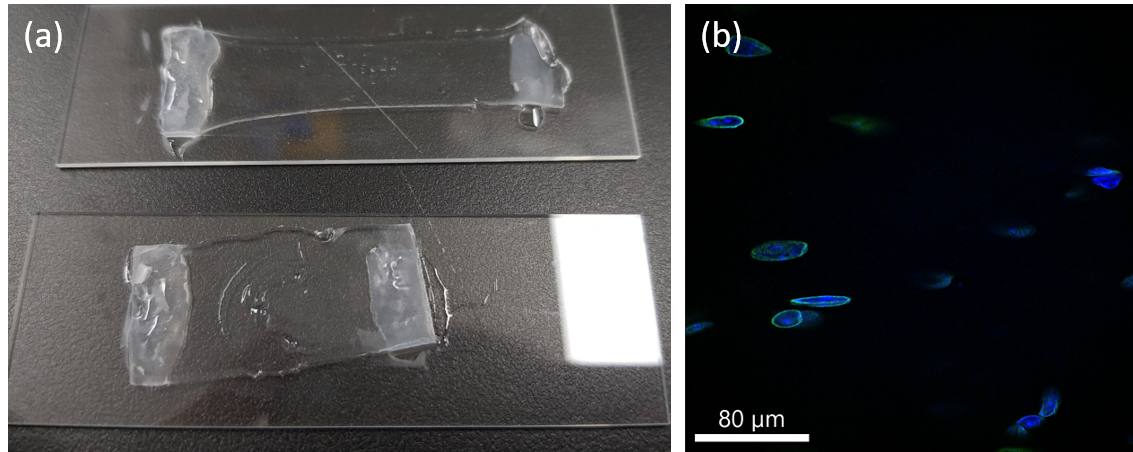


**Supplementary Figure 7.** Images of brain while stretching. (**a**) Macroscopic image of stretched brain-hydrogel complex (up) and unstretched brain-hydrogel complex (down). (**b**) Microscopic image of stretched brain-hydrogel complex. Green: Lamin B1, Blue: DAPI. Microscopic images were obtained with 40× water immersion objective lens, 1.15 NA

| **Material list** | | |
| --- | --- | --- |
| **Product name** | **Vendor** | **Product number** |
| **Cell culture and preparation** | | |
| BS-C-1 | Korean Cell Line Bank | 10026 |
| MEM (Minimum Essential Medium) | Gibco | 11095098 |
| Penicillin-Streptomycin (10,000 U/mL) | Gibco | 15140122 |
| Sodium pyruvate (100mM) | Gibco | 11360070 |
| Fetal Bovine Serum | Gibco | 10082147 |
| Phosphate-Buffered Saline 10X (PBS) | Invitrogen | AM9625 |
| Paraformaldehyde 16% Aqueous Solution | Electron Microscopy Sciences | 15710 |
| Glutaraldehyde 8% Aqueous Solution | Electron Microscopy Sciences | 16019 |
| Sodium borohydride | Sigma-Aldrich | 213462 |
| **Cell staining** | | |
| Triton X-100 | Sigma-Aldrich | X-100 |
| Normal goat serum | Jackson Immunoresearch | 055-000-121 |
| **Cell gelation and expansion** | | |
| Acryloyl-X, SE, 6-((acryloyl)amino)hexanoic Acid, Succinimidyl Ester (AcX) | Invitrogen | A20770 |
| Acrylamide (AAm) | Sigma-Aldrich | A9099 |
| Sodium acrylate (NaAA) | Ambeed | A107105 |
| *N,N’*-Methylenebisacrylamide (BIS) | Sigma-Aldrich | M7279 |
| Ammonium persulfate (APS) | Sigma-Aldrich | A3678 |
| *N,N,N’,N’*-Tetramethylethylenediamine (TEMED) | Sigma-Aldrich | T7024 |
| 4-hydroxy-TEMPO (H-TEMPO) | Sigma-Aldrich | 176141 |
| Proteinase K | New England Biolabs | P8107S |
| Ethylenediaminetetraacetic acid (EDTA) | Sigma-Aldrich | EDS |
| Trizma hydrochloride solution | Sigma-Aldrich | T3038 |
| Sodium chloride | Sigma-Aldrich | 71376 |
| **Antibody – Fluorophore conjugation and purification** | | |
| Sodium bicarbonate | Sigma-Aldrich | S6297 |
| AffiniPure Donkey Anti-Guinea Pig IgG (H+L) | Jackson ImmunoResearch | 706-005-148 |
| AffiniPure Donkey Anti-Rabbit IgG (H+L) | Jackson ImmunoResearch | 711-005-152 |
| NAP-5 50 ST | Cytiva | 17-0853-02 |
| Amicon Ultra-0.5 Centrifugal Filter Unit | Amicon | UFC505096 |
| **Brain preparation** | | |
| Isoflurane | Hana Pharm Co. | 657801261 |
| 4% Paraformaldehyde Solution | T&I | BPP-9004 |
| Glycine | Sigma-Aldrich | 50046 |
| Sodium azide | Sigma-Aldrich | S2002 |
| **Brain gelation and denaturation** | | |
| Sodium dodecyl sulfate | Sigma-Aldrich | L3771 |
| Sodium sulfite | Sigma-Aldrich | S0505 |
| **Sample mounting** | | |
| Poly-L-Lysine Solution | Sigma-Aldrich | A-005-M |

**Supplementary Table 1**. Materials used in this study

| **Primary antibody list** | | | | |
| --- | --- | --- | --- | --- |
| **Product name** | **Vendor** | **Product number** | **Stock ratio** | **Dilution ratio** |
| **Cell staining** | | | | |
| Rabbit anti-beta tubulin | Abcam | Ab6046 | 1 mg/mL | 1:200 |
| **Brain staining** | | | | |
| Rat anti-Histon H3 | Abcam | Ab10543 | 0.6 mg/mL | 1:250 |
| Rabbit anti-vGluT1 | Synaptic Systems | 135 303 | 1 mg/mL | 1:500 |
| Chicken anti-vGluT2 | Synaptic Systems | 135 416 | 1 mg/mL | 1:500 |
| Rabbit anti-SLC2A1 | Atlas Antibodies | HPA031345 | 0.4 mg/mL | 1:1000 |
| Rat anti-MBP | Abcam | Ab7349 | 1 mg/mL | 1:500 |
| Rabbit anti-Synaptophysin | Abcam | Ab32127 | 0.25 mg/mL | 1:500 |
| Mouse anti-CCP | Invitrogen | MA1-065 | 6 mg/mL | 1:500 |
| Rat anti-tubulin | Abcam | Ab6160 | 1 mg/mL | 1:500 |
| Rabbit anti-CNP1 | Synaptic Systems | 355 002 | 1 mg/mL | 1:500 |
| Rat anti-SOX2, Alexa Fluor™ 488 | Invitrogen | 53-9811-82 | 0.5 mg/mL | 1:1000 |
| Rabbit anti-Calretinin | Abcam | Ab702 | 1 mg/mL | 1:100 |
| Mouse anti-ATPB | Abcam | Ab14730 | 1 mg/mL | 1:1000 |
| Mouse anti-TOMM20 | Abcam | Ab56783 | 1 mg/mL | 1:1000 |
| Rabbit anti-Parvalbumin | Novus Biologicals | NB120-11427 | 1 mg/mL | 1:1000 |
| Rabbit anti-VAMP2 | Abcam | Ab3349 | 1 mg/mL | 1:1000 |
| Chicken anti-MAP2 | Abcam | Ab3347 | 1 mg/mL | 1:2000 |
| Chicken anti-Vimentin | Millipore | AB5733 | 1 mg/mL | 1:1000 |
| Rabbit anti-GAD 65/67 | Millipore | AB1511 | 1 mg/mL | 1:1000 |
| Chicken anti-GFAP | Aves labs | GFAP | 2 mg/mL | 1:2000 |
| Rabbit anti-Lamin B1 | Abcam | Ab16048 | 1 mg/mL | 1:2000 |
| Rabbit anti-CALB1 | Atlas Antibodies | HPA023099 | 0.3 mg/mL | 1:1000 |
| Mouse anti-Lamin A/C | Cell Signaling | 4777S | 1 mg/mL | 1:200 |
| Rabbit anti-ABAT | Atlas Antibodies | HPA041690 | 0.1 mg/mL | 1:2000 |
| Guineapig anti-MAP2 | Synaptic Systems | 188 004 | 1 mg/mL | 1:500 |
| Mouse anti-MTCO1 | Abcam | Ab14705 | 1 mg/mL | 1:200 |
| Rabbit anti-CALB2 | Atlas Antibodies | HPA007305 | 1.1 mg/mL | 1:1000 |
| Guineapig anti-Giantin | Synaptic Systems | 263 004 | 1 mg/mL | 1:500 |
| Mouse anti-Bassoon | Enzo Life Sciences | ADI-VAM-PS003-F | 1 mg/mL | 1:400 |
| Rabbit anti-Homer 1 | Synaptic Systems | 160 003 | 1 mg/mL | 1:500 |
| Guineapig anti-Piccolo | Synaptic Systems | 142 104 | 1 mg/mL | 1:200 |
| Goat anti-Collagen Type Ⅳ | Millipore | AB769 | 1 mg/mL | 1:40 |
| **Secondary antibody list** | | | | |
| **Cell staining** | | | | |
| DAPI | Sigma-Aldrich | D9542 | 1mg/mL | 1:400 |
| Goat anti-rabbit Alexa Fluor 546 | Invitrogen | A-11035 | 2mg/mL | 1:400 |
| **Brain staining** | | | | |
| DAPI | Sigma-Aldrich | D9542 | 1mg/mL | 1:400 |
| Lectin, DyLight™ 649 | Vector Laboratories | DL-1178-1 | 1 mg/mL | 1:1000 |
| Goat anti-Rat, Alexa Fluor™ 488 | Thermo Fisher | A11006 | 2 mg/mL | 1:500 |
| Goat anti-Rabbit, Alexa Fluor™ 546 | Thermo Fisher | A11035 | 2 mg/mL | 1:500 |
| Goat anti-Chicken, CF®633 | Biotium | 201261 | 2 mg/mL | 1:500 |
| Donkey anti-Rabbit, Alexa Fluor™ 488 | Manually conjugated | - | - | 1:500 |
| Donkey anti-Sheep, Alexa Fluor™ 546 | Thermo Fisher | A21098 | 2 mg/mL | 1:500 |
| Donkey anti-Mouse, Alexa Fluor™ 546 | Thermo Fisher | A10036 | 2 mg/mL | 1:500 |
| Donkey anti-Guineapig, Alexa Fluor™ 546 | Manually conjugated | - | - | 1:500 |
| Donkey anti-Rat, CF®633 | Biotium | 20137 | 2 mg/mL | 1:500 |
| Donkey anti-Rabbit, CF®633 | Biotium | 20125 | 2 mg/mL | 1:500 |
| Donkey anti-Chicken, CF®633 | Biotium | 20168 | 2 mg/mL | 1:500 |
| Donkey anti-Goat, CF®633 | Biotium | 20127 | 2 mg/mL | 1:500 |
| Donkey anti-Guineapig, CF®633 | Biotium | 20171 | 2 mg/mL | 1:500 |

**Supplementary Table 2**. Antibody list used in this study.

| **Target (available on ExELAST)** | **Cat #** | **Target (not available on ExELAST)** | **Cat #** |
| --- | --- | --- | --- |
| vGluT1 | 135 303 | Histone H3 | Ab10543 |
| SLC2A1 | HPA31345 | vGluT2 | 135 416 |
| Lectin 649 | DL-1178-1 | CCP | MA1-065 |
| MBP | ab7349 | ATPB | ab14730 |
| Tubulin | ab6160 | Parvalbumin | NB120-1142 |
| CNP1 | 355 002 | VAMP2 | ab3347 |
| SOX2(conjugated) | 53-9811-82 | Lamin A/C | 4777S |
| Claretinin | ab702 | Giantin | 263 004 |
| MAP2 | Ab5392, 188 004 | Synaptophysin | 104 202 |
| GAD65/67 | AB1511 | TOMM20 | Ab56783 |
| Vimentin | AB5733 |  |  |
| Lamin B1 | ab16048 |  |  |
| GFAP | GFAP |  |  |
| ABAT | HPA041690 |  |  |
| CALB2 | HPA007305 |  |  |
| Homer | 160 033 |  |  |
| Bassoon | ADI-VAM-PS003-F |  |  |
| Piccolo | 142 104 |  |  |
| CALB1 | HPA023099 |  |  |
| MTCO1 | Ab14705 |  |  |

**Supplementary table 3**. Antibody screening test for ExELAST. All the images were shown on supplement figure 4.
